# High peak drinking levels mediate the relation between impulsive personality and injury risk in emerging adults

**DOI:** 10.1186/s40621-024-00487-4

**Published:** 2024-02-13

**Authors:** Fakir Md. Yunus, Catherine Standage, Chantal Walsh, Peri Lockhart, Kara Thompson, Matthew Keough, Marvin Krank, Allyson Hadwin, Patricia J. Conrod, Sherry H. Stewart

**Affiliations:** 1 Department of Psychology and Neuroscience, Dalhousie University, Halifax, NS B3H 4R2 Canada; 2 Department of Clinical Psychology, University of Prince Edward Island, Charlottetown, PE C1A 4P3 Canada; 3 Injury Free Nova Scotia, Halifax, NS B3K 0E4 Canada; 4 Department of Psychology, St. Francis Xavier University, Antigonish, NS B2G 2W5 Canada; 5 Department of Psychology, York University, Toronto, ON M3J 1P3 Canada; 6 Department of Psychology, University of British Columbia, Kelowna, BC V1V 1V7 Canada; 7 Department of Educational Psychology and Leadership Studies, University of Victoria, Victoria, BC V8P 5C2 Canada; 8 Department of Psychiatry and Addictology, Université de Montréal, Montréal, QC H3T 1J4 Canada; 9 Department of Psychiatry, Dalhousie University, Halifax, NS B3H 2E2 Canada

**Keywords:** Physical injury, Personality traits, Substance misuse, Alcohol, Emerging adults

## Abstract

**Background:**

Alcohol-induced injury is one of the leading causes of preventable morbidity and mortality. We investigated the relationship between impulsive personality and physical injury (e.g. falls, sports), and whether peak drinking quantity specifically, and/or risky behaviour more generally, mediates the relationship between impulsivity and injury in undergraduates.

**Method:**

We used data from the winter 2021 UniVenture survey with 1316 first- and second-year undergraduate students aged 18–25 years (79.5% female) from five Canadian Universities. Students completed an online survey regarding their demographics, personality, alcohol use, risky behaviours, and injury experiences. Impulsivity was measured with the substance use risk profile scale, past 30-day peak alcohol use with the quantity-frequency-peak Alcohol Use Index, general risky behaviour with the risky behaviour questionnaire, and past 6-month injury experience with the World Health Organization’s (2017) injury measurement questionnaire.

**Results:**

Of 1316 total participants, 12.9% (*n* = 170) reported having sustained a physical injury in the past 6 months. Mean impulsivity, peak drinking quantity, and risky behaviour scores were significantly higher among those who reported vs. did not report injury. Impulsivity and peak drinking quantity, but not general risky behaviour, predicted injury in a multi-level generalized mixed model. Mediation analyses supported impulsivity as both a direct predictor of physical injury and an indirect predictor through increased peak drinking (both *p* < .05), but not through general risky behaviour.

**Conclusion:**

Results imply emerging adults with impulsive tendencies should be identified for selective injury prevention programs and suggest targeting their heavy drinking to decrease their risk for physical injury.

**Supplementary Information:**

The online version contains supplementary material available at 10.1186/s40621-024-00487-4.

## Background

Injury in any form remains a major public health concern for young people globally. The most recent 2019 Global Burden of Disease (GBD) study ranked road injuries as the top cause of disability-adjusted life years (DALYs) among both adolescents (10–24 years) and young adults (25–49 years) (Abbafati et al. 2020). Other common forms of physical injury such as self-harm and interpersonal violence ranked as the third and fifth top causes of DALYs, respectively, among these younger age groups (Abbafati et al. 2020). The estimated total lifetime costs of injuries (medical and productivity loss) for adolescent and emerging adults (15–24 years) in the USA in 2019 was $512,206 million USD (Peterson et al. 2021). One of the reasons that the younger age groups are particularly susceptible to many forms of physical injury may be due to their increased susceptibility to heavy drinking and other forms of risk taking.

The relationship between alcohol use and physical injury has been well established in the literature. In fact, alcohol consumption has been found to be one of the prime causes of physical injury (Borges et al. 2006; Cherpitel et al. 2003; Cremonte and Cherpitel 2014; Watt et al. 2006). Nearly half of all deaths due to alcohol globally (i.e. 45.7%) involved physical injury; unintentional and intentional injuries involving alcohol each contributed 32.0% and 13.7%, respectively, to global mortalities (World Health Organization 2007a). Emerging adults (18–25 years) in particular (Arnett 2000), may be susceptible to alcohol-related physical injury since heavy episodic drinking (HED, i.e. 5 + drinks on a single occasion in the past 30 days) reaches its peak in this developmental phase (Hingson et al. 2017; Petker et al. 2019). For example, 41.0% and 38.8% of Canadian and American emerging adults, respectively, reported weekly HED (Hingson et al. 2017; Petker et al. 2019). Other studies have shown that within the first 6 h of alcohol consumption, emerging adults have a fivefold increased risk of injury relative to the sober state (Borges et al. 2013; Williams et al. 2011), with the risk much higher for females (McLeod et al. 1999; Stockwell et al. 2002). This gender difference may be because females reach a higher blood alcohol concentration after consuming the same volume of alcohol as males due to biologically based sex differences in alcohol metabolism (Frezza et al. 1990; Taylor et al. 1996).

However, the risk for injury associated with alcohol use also varies across drinking practices, exposure, and contexts (Cremonte and Cherpitel 2014)..HED (earlier known as “binge drinking”) is defined as consuming 5 or more drinks (in adult males) or 4 or more drinks (in adult females) [which corresponds to a peak blood alcohol concentration of ~ 0.08% or more] on the same occasion or within a couple of hours, on at least 1 day in the past month (National Institute on Alcohol Abuse and Alcoholism (NIAAA) 2022). Recent USA statistics reported that 54.3% of emerging adults consumed alcohol in the past month and 34.3% engaged in HED according to the NIAAA definition (National Survey of Drug Use and Health 2020). Compared to younger (12–17 years) and older (> = 26 years) cohorts, emerging adults reported the highest prevalence (8.4%) of frequent past month heavy drinking (i.e. HED on 5 or more days) (National Survey of Drug Use and Health 2020). The high prevalence of heavy drinking practices among emerging adults poses an increased risk for experiencing alcohol induced physical injury in this demographic group.

The onset of alcohol intake, its continuation, HED and alcohol dependence are all significantly influenced by the personality trait of impulsivity (Courtney et al. 2012; Herman and Duka 2019). Impulsivity is generally understood as a decreased capacity to control behaviour in the face of reward or punishment cues, and a tendency to act without sufficient forethought (Woicik et al. 2009). Research on alcohol use disorder aetiology focusing on the role of personality has been repeatedly directed towards impulsive traits (Shin et al. 2012). Impulsivity has been linked to a higher likelihood of risk-taking behaviour across a range of domains, including health-influencing behaviours which may result in developing obesity, cardiovascular risk, and alcohol and other substance misuse (Herman et al. 2018). Such risk-taking behaviours associated with impulsivity may in turn make people more susceptible to physical injury (Pickett et al. 2006). For instance, risk taking behaviours such as using alcohol or other drugs, and HED, have been identified as risk factors for injury in young people (Galambos and Tilton-Weaver 1998; Koven et al. 2005; Pickett et al. 2002a, b).

Behavioural enactment theory suggests two systems that control our reactions, the reflective and the impulsive systems (Metcalfe and Mischel 1999). Behaviour driven by the reflective system is knowledge based with consideration of the consequences of actions; in contrast, behaviours driven by the impulsive system are more spontaneous and automatic reactions (Strack et al. 2004). Studies have suggested that those under the influence of alcohol rely on the impulsive system due to an acute alcohol-induced breakdown in their reflective system, thereby leading to risky behaviours while under the influence of alcohol (Jakubczyk et al. 2013b). It has also been suggested that individuals high in the trait of impulsivity are less likely to rely on the reflective than the impulsive system, thereby leading to increases in risky behaviour (Jakubczyk et al. 2013b). These risky behaviours can in turn result in physical injury among impulsive individuals. An important next step in this line of research would be to consider heavy drinking specifically and risk taking more generally as mediators (i.e. explanatory, intervening variables) of the relationship between impulsivity and physical injury. Given the previous evidence of associations between impulsivity, alcohol consumption, risk-taking, and injury with one other, we investigated the link between impulsivity and physical injury in emerging adults and tested whether peak drinking and/or risk-taking behaviour more generally mediate the relationship between impulsivity and physical injury among emerging adults. We hypothesized that impulsivity would be positively linked with physical injury (*H*_1_), and that both peak drinking specifically (*H*_2_) and risk-taking more generally (*H*_3_) would independently mediate the relationship between impulsivity and physical injury.

## Methods

### Participants

A cross-sectional self-report survey was carried out online in January–April 2021 across five Canadian universities (Dalhousie University, Saint Francis Xavier University, York University, Université de Montréal, and University of British Columbia-Okanagan) as a part of the broader UniVenture project (Lambe et al. 2022; Morris et al. 2022; Thibault et al. 2022; Yunus et al. 2022). Emerging adults aged between 18 and 25 years of age (Arnett 2000) were eligible if they were registered as undergraduate students (either part-time or full-time) in their 1st or 2nd year of study at the time of the survey at one of the five participating universities.^1^ A total of 2441 students started the online survey with 2060 students completing the survey, resulting in a study attrition rate of 15.6% (Yunus et al. 2022). Finally, individuals who did not meet the eligibility (age 18–25 years, first and second year of study) were excluded from the dataset and a total of 1316 students’ cleaned data were analysed. Of this final sample, 170 [12.8%] reported having experienced physical injury in the past six months.

### Measures

We used the Substance Use Risk Profile Scale (SURPS; Woicik et al. 2009**)**—a well-established and validated 23-item self-report scale of the four-factor model of personality vulnerability to substance misuse. Consistent with the usual use of the SURPS (Woicik et al. 2009), no specific timeframe was specified since such personality traits are persistent and stable over time (DeYoung and Rueter 2010). The SURPS assesses four distinct personality traits [i.e. anxiety sensitivity (AS), hopelessness (HOP), sensation seeking (SS), and impulsivity (IMP)] and having any of these traits places an individual at higher risk for substance use and related problems (Conrod 2016; Newton et al. 2016a; Woicik et al. 2009). Although the SURPS was originally designed as a measure of personality risk for substance misuse, the traits tapped by its subscales are independently valid for identifying emotional and behavioural problems (Conrod 2016; Newton et al. 2016a; Woicik et al. 2009). Moreover, none of SURPS items generally (nor impulsivity items specifically) include substance use as part of the behaviours assessed.

In the present study, we focused on the 5-item IMP subscale as the measure of impulsivity traits. The SURPS impulsivity subscale asked participants to indicate the extent to which they agree with a set of statements describing impulsivity traits (e.g. “*I often involve myself in situations that I later regret being involved in”; “I usually act without stopping to think”*). Responses were collected on a 4-point Likert scale (1 = strongly disagree; 2 = disagree; 3 = agree; 4 = strongly agree). A total score was calculated by summing the responses to the five items (Woicik et al. 2009) with a possible range of 5–20. This subscale has been validated in several languages (Jurk et al. 2015; Kaminskaite et al. 2020; Long et al. 2018). The internal consistency (Cronbach’s alpha [*α*]) of the impulsivity scale in the present sample was acceptable at *α* = 0.70.

We used the Quantity Frequency Index (QFI; Dimeff et al. 1999) to assesses their quantity of drinking on their heaviest drinking occasion in the past month (i.e. their “peak” drinking occasion). Specifically, respondents were asked “*How many drinks did you consume on the occasion that you drank the most during the last 30 days?”* (Dimeff et al. 1999)*.* Several earlier studies used this measure to determine students’ peak drinking behaviours (e.g. Baer et al. 2001; Collins et al. 2002; Dimeff et al. 1999). We selected peak drinking as our alcohol use index of interest since several prior studies reported that infrequent drinkers who consumed large amounts on single occasions are more likely to experience adverse outcomes than those who drink the same overall amount of alcohol but more frequently and in lower doses (Baer et al. 2001; Collins et al. 2002; Dimeff et al. 1999; Wechsler and Nelson 2001). Participants’ propensity towards risk taking was measured using the validated 9-item Risky Behaviour Questionnaire (RBQ; Weiss et al. 2018). This measure assesses the frequency of past year clinically relevant risk-taking behaviours (Weiss et al. 2018). The RBQ is comprised of 9-items tapping a variety of risk-taking behaviours such as driving under the influence of alcohol, having unprotected sex or sex with a stranger, damaging or destroying property, using cannabis or cocaine, shoplifting, and driving over the speed limit. For example, items were phrased as follows: *“For each of the following types of behaviour, indicate how many times you have participated in it during the past year: …Driven while under the influence of alcohol… Driven a car at over 130 kms per hour… Had sex with someone you didn't know well…”.* Each item was rated on a 5-point Likert scale of past year frequency (i.e. 0 times [scored as 1], Once [scored as 2], 2–5 times [scored as 3], 6–10 times [scored as 4], and more than 10 times [scored as 5]) and summed across the nine items. Internal consistency of the RBQ in the current sample was acceptable (*α* = 0.78).

A dichotomous measure of having experienced [or not experienced] physical injury in the past 6 months was the study outcome variable *(i.e. Have you suffered an injury/injuries in the last 6 months?).* We adopted the definition of injury from the World Health Organization (WHO) Collaborative Study on Alcohol and Injuries, and it was provided to participants in the questionnaire. Injury was defined as follows: *“Injuries are caused by acute exposure to physical agents such as mechanical energy, heat, electricity, chemicals, and ionising radiation interacting with the body in amounts or at rates that exceed the threshold of human tolerance. In some cases (for example, drowning and frostbite), injuries result from the sudden lack of essential agents such as oxygen or heat.”* (World Health Organization 2007b).

### Procedure

Data were collected online using the REDCap (Research Electronic Data Capture) survey tool (Harris et al. 2019). Since one site was a predominantly French speaking institution, we used a translated version of the English survey at that site. We provided $15 CAD or a 0.5 SONA credit point towards a grade in a participating course (at applicable sites) as compensation for completing the survey. Offering partial course credit as compensation for participation in educational research experiences is common for university student participants (e.g. Crosby and Witte 2023; MacKay and DeCicco 2020; Mason and Mullins-Sweatt 2021). As approved by the Research Ethics Boards at the five participating university sites, we provided credit points towards a final grade in a participating course as compensation for the time and effort students invested in completing the survey. Participants had to provide their institutional email address to receive their compensation; emails were stored separately from the data to protect participants’ confidentiality. Each participant was able to complete the survey only once. The entire survey took ~ 45 min to complete.

### Statistical analysis

SPSS version 27 (Chicago, IL) was used for data cleaning, coding-recoding, organization, and to run descriptive statistics. We coded study site as 1–5 (i.e. site 1, site 2, etc.) to maintain the confidentiality of the various participating post-secondary institutions. We used jamovi (v 2.0; The jamovi project 2021) to run multi-level generalized mixed models (Logistic) to estimate both within-group and between-group variability in the study variables through maximum likelihood estimation (MLM) where missing values were assumed to be missing at random. The outcome variable was dichotomous (past 6-month physical injury experience) with age, biological sex, impulsivity, peak drinking quantity, and general risky behaviour as fixed effects, and study site as a random effect. We constructed four mixed models for additional insight into the relationship between the predictors and outcome variable adjusted for age and participant’s biological sex. Model I estimated the null model [unconditional effect of the random effect variable (i.e. study site) on the outcome]. Model II was a partial model comprised of impulsivity and risk-taking behaviour (without the peak drinking variable). A similar partial model (Model III) was constructed that was comprised of impulsivity and peak drinking without the risky behaviour variable. Model IV was the full model comprised of all predictor variables (i.e. impulsivity, peak drinking, and risky behaviour). Model II and Model III separate out effects of impulsivity and risk-taking behaviour, and impulsivity and peak drinking, respectively, providing a better understanding of their unique contributions to the outcome of injury risk. Proportional change in variance (PCV) and Akaike information criterion (AIC) were calculated for each model to capture the changes in predicted variance across models and to identify the best-fitting model.

With respect to the mediational model, since both peak drinking quantity and general risk-taking behaviour were significantly correlated with impulsivity and also had significant independent relationships with the outcome variable (injury yes/no) in the mixed model, we ran one single mediation model using the SPSS PROCESS Macro version 3.5 (Hayes 2022) where impulsivity was treated as the predictor, peak drinking quantity and general risk-taking behaviour were treated as simultaneous mediators, and past 6-month injury experience (yes/no) was treated as the outcome variable. The mediational model controlled for age, sex, and study site. We used 5000 bootstrapped samples to establish bias-corrected 95% confidence intervals to estimate indirect effects in the mediation analysis. Significance levels were set a priori at *p* < 0.05.

## Results

Descriptive statistics regarding the injuries sustained are presented in Table 1. Of those students who responded that they had experienced a physical injury in the past 6 months (*N* = 170), 29.7% of the injuries were sustained while participating in sport or leisure activities, and 27.0% were caused by a fall or trip. About one-third (29.9%) of the reported injuries involved a strain, sprain, or dislocation, and about a quarter (24.2%) had consumed alcohol at the time of injury. Of the total with reported physical injuries (*N* = 170), only a small percentage (3%) sought medical assistance, and around 2% visited a general practitioner.

**Table 1:** Characteristics of the reported physical injuries (*N* = 170, 12.9%)

Injury categories	*N* (%)
*Cause of injury*	
Participating in sport and leisure	77 (29.7)
Fall, trip	70 (27.0)
Hurting myself on purpose (e.g. slashing)	21 (8.1)
Stab, cut, bite	16 (6.2)
Blunt force injury	13 (5.0)
Being in a vehicle collision (when I was the driver)	10 (3.9)
Others and unknown	52 (20.1)
*Type of injury*	
Strain, sprain, dislocation	75 (29.9)
Lacerations, Bruise, scrape, superficial wound	74 (29.5)
Cut, bite, penetrating injury, open wound	26 (10.2)
Concussion, closed head injury	24 (9.4)
Breaks/fracture	23 (9.1)
Others (N 8) and unknown (N 22)	30 (11.9)

Table 2 presents the distribution of the mean (**± **SD) age, sex, and key study variables by physical injury [yes/no]. Age and biological sex distribution were not significantly different between those who had experienced physical injury in the past 6 months and those who had not. However, impulsivity, peak drinking quantity, and general risky behaviour were all significantly higher among those who reported physical injury compared to those who had experienced no physical injury in the last 6 months. Furthermore, impulsivity, peak drinking quantity, and general risk-taking behaviours were significantly and positively inter-correlated.

**Table 2:** Key study variables as a function of past 6-month injury [yes/no]

Variables	Injury			*p*	
	Yes [*n* = 170]	No [*n* = 1148]			
Mean difference^a^ (mean ± SEM)			*t*		Cohen’s d
Age	19.28 (0.01)	19.26 (0.04)	0.17	0.861	0.014
Peak drinking quantity [past 30 days]	7.52 (0.44)	6.38 (0.16)	2.6	0.009**	0.261
Impulsivity	11.40 (0.22)	10.91 (0.08)	2.22	0.026*	0.185
Risky behaviour	16.44 (0.51)	15.09 (0.18)	2.63	0.008**	0.217

Association^b^			*χ*^2^	*p*	
Sex	[*N* (%)] *				
Male	38 (2.90)	229 (17.50)	0.49	0.498	–
Female	132 (10.10)	910 (69.50)			–

Correlation^c^	(1)	(2)	(3)	–	–
Impulsivity (1)	–			–	–
Peak drinking quantity (2)	0.189**	–		–	–
Risky behaviour (3)	0.241**	0.440**	–	–	–

^a^Independent sample *t*-test

^b^Chi-square (χ^2^) test

^c^Pearson correlation (*r*) test [controlling for ‘Age’, ‘Sex’, and ‘Study site’]

Significant at *p < 0.05; **p < 0.01

Table 3 presents the multi-level generalized mixed logistic regression model. There was no study site variation (ICC 0.00%) observed across the five universities in the reporting of physical injury experiences in Model I. Model III (i.e. partial model with impulsivity and peak drinking) explained 2.43% of the variability in physical injury reported across the study site with a better model fit (AIC = 652.48) compared to all models holding the fixed variables (age, biological sex, impulsivity, peak drinking quantity, and general risky behaviour) and the random variable (study site) constant. Risk-taking was found to significantly increase the likelihood [OR 1.03 (1.00; 1.05)] of experiencing physical injury in model II (AIC = 987.61); however, we did not find a significant relationship of impulsivity with physical injury in that model. Model III suggested significantly higher odds for physical injury among those who were more impulsive and/or who reported a higher peak drinking quantity (AIC = 652.48). Model IV (AIC = 653.77) showed that 1 unit increase in peak drinking quantity was associated with a 1.092 (95% CI 1.00; 1.18) times greater likelihood of experiencing physical injury. Similarly, 1 unit increase in impulsivity scores was associated with a 1.055 (95% CI 1.00; 1.11) times greater odds of experiencing physical injury. We did not find age, biological sex, or general risky behaviours as a significant predictor of physical injury experience among the emerging adult population in the adjusted Model IV (final model). Although Model III had a slightly smaller AIC compared to Model IV indicating that Model III is a better model, we considered model IV as our final model since it is the only model that simultaneously examines both mediators and the difference in the AICs in Models III and IV were quite small (AIC = 652.48 vs 653.77, respectively).

**Table 3:** Factors associated with injury (past 6 months)

Characteristics	Model I,^a^	Model II,^b^	Model III,^c^	Model IV,^d^
	OR (95% CI)	OR (95% CI)	OR (95% CI)	OR (95% CI)
*Individual level*				
Age	–	1.017 (0.90; 1.14)	0.983 (0.83; 1.165)	0.978 (0.82; 1.16)
Sex	–			
Male	–	Ref	Ref	Ref
Female	–	0.933 (0.62; 1.39)	1.021 (0.60; 1.72)	1.014 (0.60; 1.71)
Risk-taking behaviour [past year]	–	1.03 (1.00; 1.05)*	–	1.015 (0.98; 1.05)
Impulsivity	–	1.053 (0.98; 1.21)	1.101 (1.01; 1.18)*	1.092 (1.00; 1.18)*
Peak drinking quantity [past 30 days]	–	–	1.064 (1.01; 1.11)*	1.055 (1.00; 1.11)*
*Measure of variation*				
Variance (SD)	0.00	0.00	0.82	0.00
ICC (%)	0.00	0.00	2.43	2.63
PCV (%)	Ref	0.00	0.00	0.00
*Model fit statistics*				
AIC	1015.47	987.61	652.48	653.77

^a^Model I (null model) was fitted without determinant variables

^b^Model II is adjusted for individual-level variables only without peak drinking variable

^c^Model III is adjusted for individual-level variables only without risk-taking behaviour variable

^d^Model IV is adjusted for individual-level variables (full model)

^*^Significant at *p* < 0.05

Fixed effect variables: age, sex, impulsivity, risk-taking behaviour and peak drinking quantity

Random effect variable: study site

**Fig. 1:**
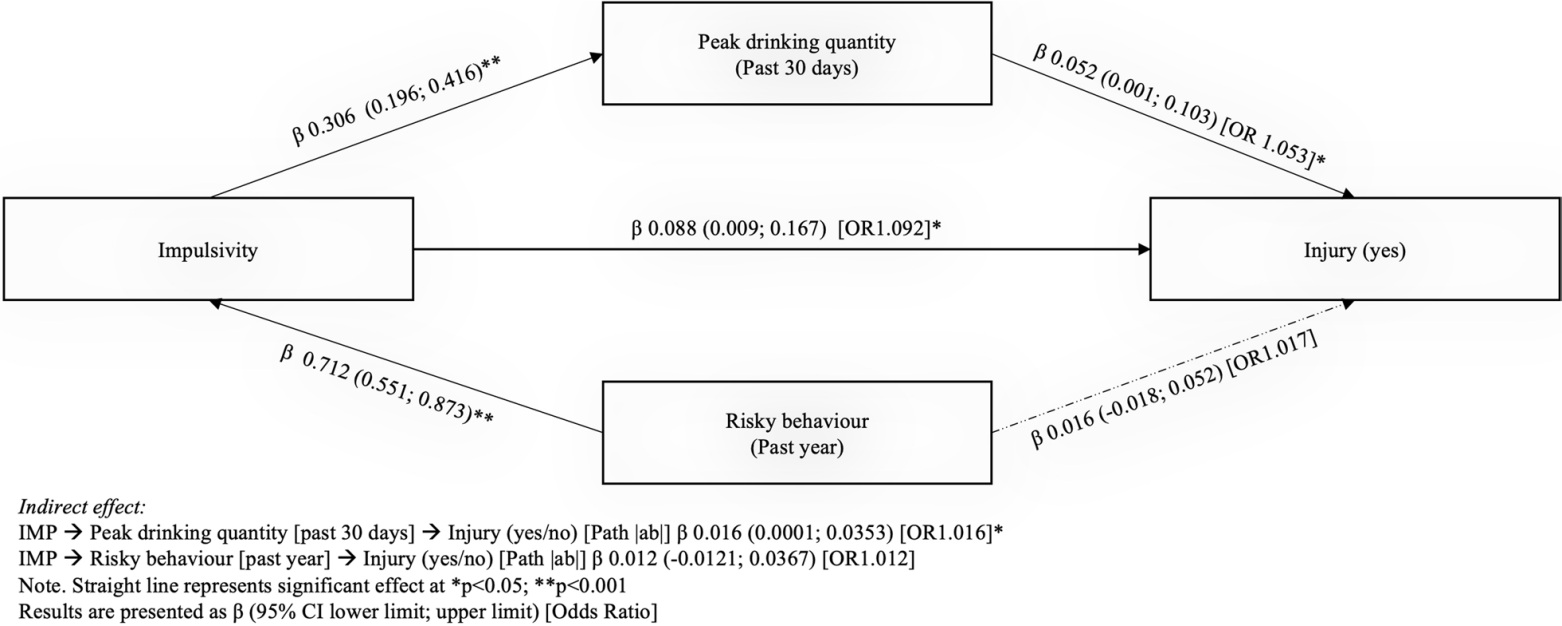
Graphical representation of simple mediation model (simultaneous model). A simple mediation diagram (simultaneous model) with unstandardized coefficients for the paths |*a*|, |*b*|, the indirect path |*ab*|, and the direct path |*ac*|, illustrates the mediating role of peak drinking quantity and general risk-taking behaviour in the relationship between impulsivity and injury experience while controlling for age, biological sex, and study site

Table 4 presents the mediation analysis covering path |*a*|, path |*b*|, the indirect path |*ab*|, and the direct path |*ac*|. For the first mediational path, we found that peak drinking quantity significantly mediated (indirect path |*ab*|) the relationship between impulsivity and injury experience after adjusting age, biological sex, study site, and general risk-taking behaviour. In addition, the direct path |ac| was also significant suggesting that impulsivity positively and directly predicted injury experience even after accounting for the pathway through peak drinking quantity. However, with respect to the hypothesized second mediator, the adjusted mediation effect of general risk-taking behaviour in explaining the link between impulsivity and injury experience was not significant (Fig. 1). As a supplementary analysis, another mediational model conducted with only the females replicated a significant indirect effect of impulsivity on injury risk through peak drinking (Additional file 1: Fig. 1). The only difference from the main analysis reported in the body of the paper was that the mediation was full rather than partial, meaning that heavy drinking fully explained the link of impulsive personality to injury risk in females. Future research with a more sex balanced sample (and a larger subsample of males) is needed to test if there are sex differences in the mediating role of peak drinking in the link of impulsivity with injury risk.

**Table 4:** Mediation effect (simultaneous model) of peak drinking quantity and general risk-taking behaviour on the association of impulsivity to injury

Predictor	Mediator	Indirect effect					Direct effect	
		Path |*a*|	Path |*b*|		Path |*ab*|		Path |*ac*|	
		(Effect of IV on M)	(Unique effect of M on DV)		(Indirect effect; IV to DV via M)		(Effect of IV on DV)	
		[*β* (95% CI)]	[*β* (95% CI)]	OR	[*β* (95% CI)]	OR	[*β* (95% CI)]	OR
Impulsivity	Peak drinking quantity [past 30 days]	0.306 (0.196; 0.416)**	0.052 (0.001; 0.103)*	1.053	0.016 (0.0001; 0.0353)*	1.016	0.088 (0.009; 0.167)*	1.092
	Risky behaviour [past year]	0.712 (0.551; 0.873)**	0.016 (− 0.018; 0.052)	1.017	0.012 (− 0.012; 0.036)	1.012		

IV: independent variable; M: mediating variable; DV: dependent variable

Dependent variable: Injury (Yes/No)

Adjusted for age, biological sex, and study site

*p < 0.05; **p < 0.001

## Discussion

This study examined the relationship of impulsivity—one of the traits in the four-factor model of personality vulnerability to substance misuse (SURPS; Woicik et al. 2009)—with physical injury among Canadian university students in their first and second year of study. The first objective of this study was to investigate if there is a significant relationship between impulsivity personality and physical injury experience (e.g. due to falls, sports). This objective helped us to understand if impulsivity personality is a predictor of injury risk. Our results suggested that impulsivity and peak drinking quantity both significantly predicted higher odds of physical injury in the adjusted model. The second objective of this study was to find out whether peak drinking quantity specifically, and/or risky behaviour more generally, mediates the relationship between impulsivity and injury in undergraduates. This objective helped us understand the potential mediational role of peak drinking quantity in explaining the increased odds of physical injury in more impulsive individuals, which helped us better understand how impulsivity personality exerts effects in increasing risk for physical injury. We found that the pathway from impulsivity to physical injury in emerging adults was partially mediated by peak drinking quantity, but not by risky behaviour more generally. These results have preventative implications for injury risk reduction in emerging adults. First, knowledge that impulsivity personality does present increased risk for physical injury is helpful in knowing whom to target for injury prevention efforts—namely, more impulsive emerging adults. Second, knowledge that impulsivity personality exerts its effects in increasing injury risk in part through increasing peak drinking levels suggests a mechanism that can be targeted among impulsive emerging adults—namely heavy drinking—in injury risk reduction efforts. More specifically, interventions known to reduce heavy drinking by impulsive young people, like the PreVenture substance misuse prevention/early intervention program (Barrett et al. 2015; Conrod et al. 2013) may be helpful in reducing impulsive emerging adults’ risk for physical injury. Our results showed that impulsivity and peak drinking quantity significantly predicted higher odds of physical injury, and that the pathway from impulsivity to physical injury in emerging adults was partially mediated by peak drinking quantity. These cross-sectional results, although limited by their inability to firmly establish directionality or causation, provided data consistent with a significant mediational role of heavy drinking in explaining the link of impulsivity with injury risk.

Although several earlier studies established the relationship between alcohol consumption and physical injury (Borges et al. 2004; Kuendig et al. 2015; Rehm et al. 2003; Watt et al. 2004), our study extends this prior work by additionally looking at whether this link of alcohol consumption to physical injury might help explain why impulsive people are at increased risk of physical injury. Moreover, general risky behaviour was included in the model as a potential alternative or additional mediator since earlier research established a link between risky behaviour and higher likelihood of physical injury (Pickett et al. 2006; Turner et al. 2004). Our findings showed that impulsive emerging adults are more likely to experience physical injury than others in part due to their specific tendency to drink more heavily than others.

We found 12.9% of the study participants had experienced one or more physical injuries in the past 6 months. Our results suggested that mean impulsivity scores, peak drinking quantity, and risky behaviour scores were significantly higher among those who reported physical injury in the past 6 months than among those reporting no physical injuries. Multi-level mixed-model analysis suggested that a higher amount consumed during one’s peak drinking episode in the last 30 days was associated with a significantly higher likelihood of experiencing physical injury after adjusting age, sex, impulsivity, and general risky behaviour.

Furthermore, our mixed model indicated a potential role of peak drinking quantity and general risk-taking behaviour in mediating the relationship of impulsivity and physical injury among emerging adults that required further exploration. Given that peak drinking quantity and general risk-taking behaviour were both correlated with impulsivity and were also significantly and independently associated with the injury outcome in the mixed model analyses, we carried out a single mediational analysis with both potential mediators entered simultaneously to find out if peak drinking specifically, and/or risk-taking behaviour generally, mediates the relationship between impulsivity and physical injury among emerging adults. Our establishment of a partial mediational relationship through peak drinking is novel since our study presents a new direction of relationship to the previously established direct relationship between alcohol consumption and physical injury and there has been no such evidence established in earlier studies. However, there are other mediators not yet accounted for in the model that could also contribute to explaining the link of impulsivity to risk for injury such as anxiety and depression. For instance, two separate studies found links between impulsivity with anxiety as well as between anxiety with an increased risk of injury, suggesting that anxiety may act as a mediator between the impulsivity and injury (Li et al. 2017; Xia et al. 2017).

Impulsive individuals have deficits in behavioural inhibition in the context of cues for reward and/or punishment (Woicik et al. 2009) that cause them to make risky choices that could place them at risk for injury. They tend to behave in ways that bring immediate reward or relief without thinking through the longer-term consequences of their behaviour—a cognitive style (Woicik et al. 2009) which could clearly place them at increased risk for injury. Moreover, individuals with an impulsivity personality are characterised by two emotion-based forms of urgency—positive and negative—which involve acting rashly in response to intense positive and negative emotions, respectively (Kaiser et al. 2016; Nguyen et al. 2013). These two aspects of trait IMP may instigate risky behaviours that place impulsivity individuals at increased risk for injury (Ryb et al. 2006). A possible neuropsychological explanation involves impairments in the functioning of the frontal lobes in impulsive individuals (Alvarez and Emory 2006). These impairments can lead to self-regulation deficits (i.e. a failure to “put on the brakes”) (Giedd 2004) which can lead to risky choices including those that increase the risk of injury.

For the very reasons noted above (e.g. difficulties with self-regulation, failure to think through longer-term consequences of their behaviour), impulsive individuals are also at risk of heavy drinking to achieve immediate reward or relief (Woicik et al. 2009). Acute alcohol intoxication further negatively impacts self-regulation (Spinola et al. 2017) due to impaired functioning of the frontal (Scaife and Duka 2009) and temporal lobes (Peterson et al. 1990), further increasing risk of physical injury. Additionally, heavy drinking can lead to acute impairment in the functioning of the reflective system (i.e. conscious, deliberative information processing), thus creating circumstances where there is less executive control over the impulsive system in determining subsequent behaviour (Banca et al. 2016; Strack et al. 2004). This would lead these individuals, when intoxicated, to behave even more automatically, putting them at risk for injury. Hence, impulsive individuals’ tendency to engage in heavy drinking could lead to alcohol-induced impairments that result in even more rash, risky choices that put them at higher risk for physical injury. It should also be acknowledged that heavy drinking also has motor and perceptual impairing effects that can lead to certain forms of physical injury (e.g. falls and motor vehicle accidents) since heavy drinking not only impairs the execution of complex motor response sequences but also inhibits pre-motor planning process (Opitz et al. 2021).

Our study found that the mean general risk-taking behaviour score was significantly higher among those who reported physical injury compared to non-injured participants. Moreover, general risk-taking behaviour significantly predicted an increased likelihood of physical injury in the model that did not adjust for peak drinking quantity. This finding replicates prior work establishing that engaging in risky behaviours increases the odds for experiencing physical injury (Pickett et al. 2006; Turner et al. 2004). However, unexpectedly, we did not find general risk-taking behaviour as a significant predictor for physical injury in the adjusted full model which controlled past month peak drinking quantity. Collinearity between peak drinking quantity and general risky behaviours was not likely at play here (VIF 1.35 and 1.37, respectively) since the inter-correlation was significant but moderate in magnitude (*r* = 0.463) (Vatcheva et al. 2016). Thus, it appears that peak drinking quantity significantly outweighs a general risk-taking tendency in terms of predicting physical injury, at least when risk-taking tendency is measured through the RBQ (Weiss et al. 2018). One potential reason for this pattern of results may because of some of the risky behaviours listed on the RBQ would have risks for physical injury (e.g. driving a motor vehicle over the speed limit) whereas others would not (e.g. engaging in unprotected sex). The unrelated items likely added noise which decreased the overall link of general risk-taking to physical injury. Our novel findings thus suggested that peak drinking quantity presents a more important risk for physical injury than a general tendency towards risk-taking behaviours. Additionally, our adjusted mediation model showed that general risk-taking behaviours did not mediate the relationship between impulsivity and physical injury after adjusting peak drinking quantity in past 30 days, and participant’s age, biological sex, and study site. While we found that higher impulsivity scores were significantly linked with increased risky behaviour, general risk taking was not significantly related to physical injury in a model that controlled peak drinking quantity (a specific form of risk-taking behaviour).

Furthermore, it is important to interpret the study results in light of the context in which the data were collected. Specifically, the study was carried out during the COVID-19 pandemic prior to the roll out of vaccinations. Alcohol consumption and risk-taking behaviour might have varied due to restrictions imposed due to the COVID-19 pandemic. For instance, various forms of COVID-19 restrictions such as social isolation (e.g. social distancing, avoidance of gatherings (even in university dorms), remote class attendance) may have negatively impacted students’ social opportunities and in turn their opportunities for drinking (e.g. 18.3% less full-service restaurants and an 18.9% reduction in drinking venues due to pandemic-related closures in Canada) (Statistics Canada 2021). Thus, the pandemic context may have led to an underestimation of the mediational role of peak drinking quantity in the impulsivity to injury relation relative to its role in non-pandemic times. However, it remains possible that university students may have used alternative (e.g. on-line) get-togethers for socializing with friends to meet and drink, a drinking context which has been associated with an increased risk for high volume alcohol use (Rubio et al. 2023). Moreover, impulsivity was found to have an inverse relationship with adherence to COVID-19 public health restrictions (Morris et al. 2022); thus, impulsive students may still have been engaging in heavy drinking despite these restrictions. Regardless, we found mediation through heavy drinking even in the context of the pandemic; it is possible that heavy drinking may be an even stronger mediator of the link of impulsivity to injury risk among students in non-pandemic times.

This study suffers from limitations which should be held in mind when interpreting the results. For example, the relationship between impulsivity and alcohol consumption may be bidirectional (Kaiser et al. 2016). While impulsivity may indeed prompt higher alcohol consumption levels, studies have shown that heavy alcohol consumption can also prompt impulsive behaviour and weaken self-regulation (Bernstein et al. 2015; Dick et al. 2010; Kaiser et al. 2016; Shin et al. 2012). Additionally, a bidirectional relationship has been established between impulsivity and non-suicidal self-injury (Hamza and Willoughby 2019). Although higher impulsivity imparts a higher risk of non-suicidal self-injury over time, non-suicidal self-injury may also impede the development of impulse control, particularly when problem coping behaviours are practiced more frequently if they are emotionally or socially rewarding and at the cost of acquiring other regulatory abilities (Fischer et al. 2004; Hamza and Willoughby 2019; Peterson and Fischer 2012). We acknowledge that yet another bidirectional relationship could have been at play between alcohol consumption and injury risk. Specifically, while it is well established that alcohol consumption increases the risk of injury (Borges et al. 2006; Cherpitel et al. 2003; Cremonte and Cherpitel 2014; Savola et al. 2005; Watt et al. 2006), certain forms of injury such as concussions leading to traumatic brain injury (TBI) have been found to be a risk factor for increased heavy drinking (Weil et al. 2018). Nonetheless, our models assumed a directional link from impulsivity to heavy drinking to physical injury. Future research should use longitudinal designs to test the directional assumptions of our models and the possibility of bidirectional relations.

Another possible limitation of our study pertains to the varying timeframes of the tested mediators, i.e. peak alcohol quantity (past 30-day) and risk-taking behaviour (past year), and the outcome (past 6-month physical injury). Considering the moderate stability of risk-taking propensity (Josef et al. 2016) and peak alcohol quantity (Reich et al. 2015), we considered our mediator measures as proxies for usual patterns of risk taking and excessive alcohol intake respectively—justifying them as potential mediators of the relations of trait impulsivity to physical injury over the past 6 months.

A further potential limitation of the study is that the study results are biased towards female respondents since the study participants were predominantly female (79.5% collapsed across sites), a sex bias which plagues survey research (Sharma et al. 2021). Therefore, the results may be more representative of female respondents’ experiences, preferences, or perspectives. However, it is important to keep in mind that there is in fact higher (57.4%) female than male enrolment in Canadian universities (Statistics Canada 2022) but not to the extreme seen in our study. Future studies may consider better matching the sample to the population from which they are being drawn in terms of sex distribution by increasing efforts to recruit male participants. Regardless, generalizability cannot be assured because data were not drawn from a nationally representative sample of first- and second-year Canadian undergraduates. However, since our data were drawn from five Canadian sites covering the east to west of Canada and including both larger and smaller universities, and urban- and rural-based universities, our study provides some degree of confidence in generalizing to Canadian undergraduates more broadly. Given that impulsivity levels are higher in males than females (Chamorro et al. 2012) and that Canadian males aged between 18 and 34 years are more likely (33.5%) to report heavy drinking than their female counterparts (23.8%) (Statistics Canada 2018), it is impressive that we still see mediation of the impulsivity to injury link through heavy drinking in our predominantly female sample. Future studies should investigate the potential moderating role of sex on this mediational relationship. Moreover, future studies might examine the potential moderating role of ethnicity since we do not know if the findings extend beyond our largely White sample (66.9% Caucasian). While personality is quite stable cross-culturally (Terracciano and McCrae 2006), it is still possible that impulsivity manifests in different types of risky behaviours in different cultural contexts (Deater-Deckard et al. 1998; Duell et al. 2018). Thus, the link of IMP to injury risk might be mediated by different risky behaviours in students from different cultural backgrounds.

Furthermore, we used a single score for impulsivity—a trait which is recognized as a multi-dimensional construct. For example, we did not distinguish between two independent and distinct aspects of impulsivity—*behavioural* (impaired ability to stop an initiated response) and *cognitive* impulsivity (impairment in weighing the consequences of one’s actions) (Arce and Santisteban 2006; Jakubczyk et al. 2013a). We call for a more nuanced assessment of impulsivity in future, so we know which aspects of impulsivity to target for injury prevention and which specific aspects of impulsivity are related to injury risk through heavy drinking. Our results must thus be interpreted as reflecting relations of impulsivity with injury susceptibility when impulsivity is conceptualized and assessed with the SURPS—namely deficits in behavioural response inhibition that are particularly visible in the context of cues for reward and/or punishment (Woicik et al. 2009).

While we relied on standardized and validated questionnaires to assess our predictor, mediators, and outcome variables, our measures all involved retrospective self-report data which are subject to several potential biases ranging from measurement bias to social-desirability bias (Rosenman et al. 2011). Future studies may consider combining the self-reported data with behavioural data or observer reports. Behavioural assessments of impulsive tendencies can provide more objective data through a variety of behavioural tasks (e.g. prepotent response inhibition, resistance to distractor interference, resistance to proactive interference, delay response, and elapsed time distortions) which depicts the person’s actual behaviour in a scenario or in response to a cue for reward or punishment, not merely what the person believes they would do (Cyders and Coskunpinar 2011; Dick et al. 2010). Nevertheless, as behavioural measures exclude social and emotional factors, they may not always reflect typical day-to-day impulsive actions (Nguyen et al. 2018).

The study results indicated that a 1 unit increase in impulsivity scores predicted a 9.2% higher odds of experiencing physical injury and that relationship was partially mediated by emerging adults’ peak drinking quantity. Our findings linking impulsivity with physical injury risk via peak drinking quantity suggests important future clinical potential of these findings for injury risk reduction in emerging adults via personality-targeted intervention. Given that impulsivity exerts its effects on injury risk through peak drinking quantity, adopting a personality targeted approach to intervention may show great promise in terms of clinical potential. For instance, a review article covering eight randomised controlled trials reviewed the current practices in personality-targeted interventions (including impulsivity focused intervention) among youth and reported moderate effects (Mean *d* = 0.47) in reducing drinking (use or frequency), binge drinking (use or frequency), and alcohol problems (presence or severity) (Conrod 2016). This personality-targeted approach is being adapted and tested for efficacy in reducing heavy drinking in university students (i.e. the UniVenture program: ClinicalTrials.gov Identifier: NCT05383989). If UniVenture is effective in reducing heavy drinking in impulsive undergraduates, given heavy drinking’s mediational role in explaining injury risk in impulsive students, personality-targeted intervention might be a good candidate for injury risk reduction efforts. Interventions tailored to impulsive personalities may help individuals drink less heavily (Newton et al. 2016b), which may in turn prevent physical injury in this group.

## Conclusions

Our results suggested that emerging adults’ impulsive traits, their peak drinking quantity and general risk-taking behaviour independently predicted a higher likelihood of experiencing physical injury holding age, biological sex, and study site variations constant. Furthermore, we found that peak drinking quantity partially mediated the significant positive relationship between impulsive personality and physical injury. Although impulsive personality was positively and independently linked with more general risk-taking behaviour and with an increased likelihood of experiencing physical injury, general risk-taking did not mediate the relationship between impulsive personality and physical injury. Our study extended the extant literature on the relationships of impulsive personality traits with peak drinking quantity, general risk-taking tendencies, and injury susceptibility, and added new knowledge on the mediating role of peak drinking quantity in helping explain the link between impulsivity and physical injury in emerging adults.

### Supplementary Information

**Additional file 1. Supplementary Figure 1:** A simple mediation diagram (simultaneous model) for female participants only. The diagram illustrates the unstandardized coefficients for the paths |a|, |b|, the indirect path |ab|, and the direct path |ac|, elucidating the mediating role of peak drinking quantity and general risk-taking behavior in the relationship between impulsivity and injury experience while controlling for age and study site.

## Data Availability

Data may be shared with qualified researchers after considering all ethical aspects and in consideration of the existing rules imposed by the REBs in question. Interested researchers may request access to the data by contacting the corresponding author.

## Abbreviations

AIC: Akaike information criterion
CCSA: Canadian centre on substance use and addiction
CRISM: Canadian research initiative in substance misuse
DALYs: Disability-adjusted life years
GBD: Global burden of disease
HED: Heavy episodic drinking
ICC: Intra-cluster correlation
IFNS: Injury free nova scotia
IMQ: Injury measurement questionnaire
MHCC: Mental Health Commission of Canada
NIAAA: National Institute on Alcohol Abuse and Alcoholism
NSHA-RMU: Nova Scotia Health Authority—research methods unit
QFI: Quantity Frequency Index
RBQ: Risky behaviour questionnaire
REDCap: Research electronic data capture
SES: Socio-economic status
SPOR: Strategy for patient-oriented research
SURPS: Substance use risk profile scale
WHO: World Health Organization

## Footnotes

1 Only first and second year students were invited to participate but it was later discovered that some third year and beyond had participated and they were excluded at the data cleaning stage.
