# Supplementary figures and images for: High peak drinking levels mediate the relation between impulsive personality and injury risk in emerging adults
Source: Inj Epidemiol. 2024 Feb 13;11:5. doi: 10.1186/s40621-024-00487-4 (PMC10863178; doi:10.1186/s40621-024-00487-4)

**Supplementary Figure -1 (Female only mediational model)**


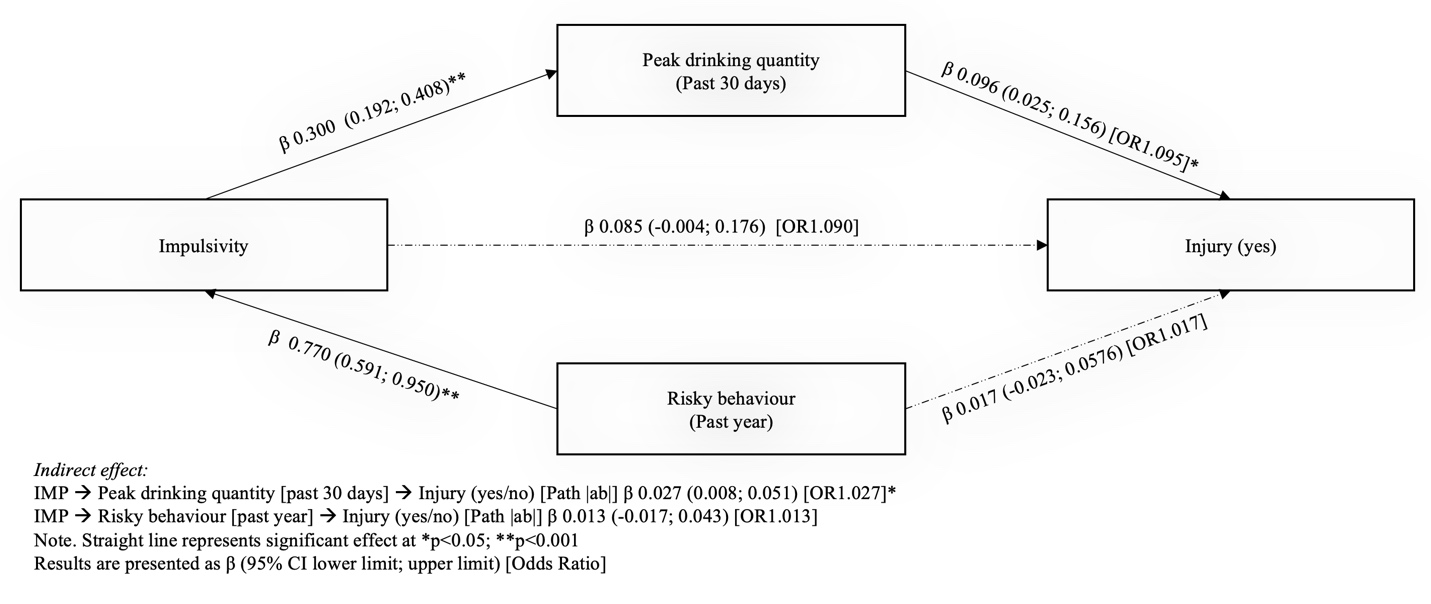

Supplement: Supplementary file 1 — Additional file 1. Supplementary Figure 1: A simple mediation diagram (simultaneous model) for female participants only. The diagram illustrates the unstandardized coefficients for the paths |a|, |b|, the indirect path |ab|, and the direct path |ac|, elucidating the mediating role of peak drinking quantity and general risk-taking behavior in the relationship between impulsivity and injury experience while controlling for age and study site. [file 40621_2024_487_MOESM1_ESM.docx]
